# The postoperative morbidity index: a quantitative weighing of postoperative complications applied to urological procedures

**DOI:** 10.1186/1471-2490-14-1

**Published:** 2014-01-03

**Authors:** Jonathan Beilan, Ruth Strakosha, Diego Aguilar Palacios, Charles J Rosser

**Affiliations:** 1 University of Central Florida College of Medicine, Orlando, FL, USA; 2 Clinical and Translational Program, University of Hawaii Cancer Center, 701 Ilalo St, Honolulu, HI 96814, USA

**Keywords:** Complication, Index, Postoperative, Quantitative, Urology

## Abstract

**Background:**

The reporting of post-operative complications in the urological field is lacking of a uniform quantitative measure to assess severity, which is essential in the analysis of surgical outcomes. The purpose of this study was to evaluate the feasibility of estimating quantitative severity weighing of post-operative complications after common urologic procedures.

**Methods:**

Using a large healthcare system’s quality database, complications were identified in eleven common urologic procedures (*e.g.,* insertion or replacement of inflatable penile prosthesis, nephroureterectomy, partial nephrectomy, percutaneous nephrostomy tube placement, radical cystectomy, radical prostatectomy, renal/ureteral/bladder extracorporeal shockwave lithotripsy (ESWL), transurethral destruction of bladder lesion, transurethral prostatectomy, transurethral removal of ureteral obstruction, and ureteral catheterization) from January 1, 2011 to December 31, 2011. Complications were classified by the Expanded Accordion Severity Grading System, which was then quantified by validated severity weighting scores. The Postoperative Morbidity Index (PMI) for each procedure was calculated where an index of 0 would indicate no complication in any patient and an index of 1 would indicate that all patients died.

**Results:**

This study included 654 procedures of which 148 (22%) had one or more complications. As would be expected, a more complex procedure like radical cystectomy possessed a higher PMI (0.267), while a simpler procedure like percutaneous nephrostomy tube placement possessed a lower PMI (0.011). The PMI of the additional nine procedures fell within the range of these PMIs. These PMIs could be used to compare surgeons, hospitals or procedures.

**Conclusions:**

Quantitative severity weighing of post-operative complications for urologic procedures is feasible and may provide exceptionally informative data related to outcomes.

## Background

The concept of outcomes measurement was first described by Dr. E. Codman in the early 1900’s and has now become a national incentive set forth by the Centers for Medicare and Medicaid Services and other organizations [1]. Although there are many well-designed programs currently in place to monitor the quality and outcomes of care in certain specialties or institutions, they tend to be complex and therefore unlikely to be effectively implemented at a national or global level [2]. One of the major outcomes to report in any surgical field is post-operative complications. The Clavien complication grading system is a severity grading system developed by Clavien *et al.,* and published in 1992. This complication grading system ranks complications based on the magnitude of the intervention(s) required for their treatment and whether the complications cause permanent injury or death [3]. In 2004, the Clavien complication grading system was modified to add more detail to the more serious complications, however, with it has come inconsistencies in the application of this grading system, *e.g*., non-uniform grade contraction [4].

As a result of these inconsistencies, the grading system was extensively modified (renamed Accordion Severity Grading System) in 2009. Specifically, the Accordion Severity Grading System added flexibility to the grading system by introducing an expandable classification, and clarity was improved by introducing rigorously defined qualitative terms [5]. However, to date, all complication severity grading systems, *e.g.,* Memorial Sloan Kettering Severity Grading System and Accordion Severity Grading System [6]. are key short-term outcomes measures of operative procedures and lack robust quantitative measure of the severity of surgical complications, which would allow comparison between two health states.

Recently, severity weighting of the Accordion Severity Grading System led to a) correction of criteria for two of the higher grades of severity and b) severity scores with weight for each of the six severity levels, enabling for the first time a way to quantify complications. Specifically, the application of severity weights to Accordion Severity Grading System (postoperative morbidity index, PMI) was applied to data gathered from American College of Surgeons (ACS) National Surgical Quality Improvement Program (NSQIP) program for the year 2007 and demonstrated that weighting of complications provided new insights into the burden contributed by specific types of complications [7,8].

Straberg *et al.* evaluated and reported the feasibility of PMI, which applied to general surgery estimates quantitative morbidity scores after surgical procedures including laparoscopic colectomy, appendectomy, and pancreaticoduodenectomy, by taking into consideration validated weighted values in post-operative complications [9]. Based on these encouraging results from our general surgery colleagues, we report on the feasibility of applying Accordion Severity Grading System and PMI in a large urologic cohort (n = 654) that underwent 11 common urologic procedures. To date the expansion of PMI as an estimation of post-operative complication of urologic procedures has not been explored.

## Methods

This study was approved by Orlando Health Inc. (Orlando, FL) Institutional Review Board with a waiver of consent. Orlando Health Inc. is a large healthcare system (> 1,000 beds) comprised of eight facilities in central Florida, affiliated with the University of Central Florida College of Medicine and Florida State University School of Medicine. Complications identified within the American College of Surgeons National Surgical Quality Improvement Program (ACS-NSQIP) within the Department of Urology in Orlando Health Inc. were queried retrospectively to gather information regarding patient outcomes to urologic surgeries performed from January 1, 2011 to December 31, 2011. Based on the number of procedures performed in 2011, the location of the procedures (in-patient and outpatient) and the difficulty of the procedures, CPT codes associated with 11 diverse procedures covering a wide range of urologic procedures [*e.g.,* insertion or replacement of inflatable penile prosthesis, nephroureterectomy, partial nephrectomy, percutaneous nephrostomy tube placement, radical cystectomy, radical prostatectomy, renal/ureteral/bladder extracorporeal shockwave lithotripsy (ESWL), transurethral destruction of bladder lesion, transurethral prostatectomy, transurethral removal of ureteral obstruction, and ureteral catheterization] were queried and included for analysis. All patients identified in each of the 11 procedures were evaluated (*i.e.,* no patient was excluded from analysis). To establish the true PMI of a procedure in an institution one would expect that >25 patients per group would be needed although the study has not yet been done to determine the exact number.

Individual medical records of the patients who underwent the above procedures were reviewed to determine the incidence of post-operative complications as defined by American College of Surgery National Surgical Quality Improvement Program (ACS NSQIP) within 30 days (any NSQIP 30-day morbidity). The ACS NSQIP complications that were noted included bleeding, superficial wound infection, deep wound infection, organ space infection, wound dehiscence, acute renal failure, progressive renal insufficiency, urinary tract infection, prolonged ileus, pneumonia, failure to wean from ventilator, unplanned intubation, pneumothorax, pulmonary embolus, cardiac arrest, exacerbation of heart failure, deep venous thrombosis, cerebrovascular accident, transient ischemic attack, sepsis, septic shock, and death (all-cause 30-day mortality). The severity of each complication was graded independently by two clinicians (JAB and RS) according to the recently validated Accordion Severity Grading System (Table 1). A third investigator (CJR) reviewed discrepancies and rendered a final score. In cases with multiple ACS NSQIP complications, the case was assigned a grade corresponding to the highest graded complication.

**Table 1:** Accordion classification system with severity weights

**Grade**	**Description**	**Severity weight**
**1**	Treatment of complication requires only minor invasive procedures that can be done at the bedside, such as insertion of intravenous lines, urinary catheters, and nasogastric tubes, and drainage of wound infections. Physiotherapy and antiemetics, antipyretics, analgesics, diuretics, electrolytes, and physiotherapy are permitted.	0.110
**2**	Complication requires pharmacologic treatment with drugs other than such allowed for minor complications, e.g. antibiotics. Blood transfusions and total parenteral nutrition are also included.	0.260
**3**	No general anesthesia is required to treat the complication: requires management by an endoscopic, interventional procedure, or reoperation without general anesthesia.	0.370
**4**	General anesthesia is required to treat complication. Alternately, single-organ failure has developed.	0.600
**5**	General anesthesia is required to treat complication and single organ failure has developed. Alternately, multisystem organ failure (2 or more organ systems) has developed.	0.790
**6**	Postoperative death occurred.	1.000

Next, a weighted postoperative morbidity index (PMI) was calculated as previously described [8] (*i.e.,* to calculate the PMI for each operative procedure, the weights of all the complications for all patients who underwent the corresponding procedure were summed and divided by the total number of patients undergoing that procedure). A PMI of 0 would indicate that no patient having the procedure had any postoperative complications, while on the other hand, and a PMI of 1.000 would indicate that every patient having the procedure suffered postoperative death. In order to analyze complication severity, the sum of severity weights for all patients having any complication after a procedure were divided by the total number of patients with complications in the group (*i.e.,* the denominator was the number of patients having a complication after the procedure, rather than the total number of patients having the procedure). Descriptive statistics were performed in Excel 2007 (Microsoft Corp).

## Results

Of the 11 procedures queried for inclusion into this study, a total of 654 corresponding surgical procedures performed by 25 attending physicians were identified. Table 2 (Additional file 1: Figure S1A) shows the number of cases and severity grade of each complication by procedure. Of the 654 surgical procedures, 506 procedures did not have an associated complication noted, thus 148 procedures were noted to be associated with a post-operative complication. Grade one complications were the most common (47%). There were no perioperative deaths (grade 6) reported. It is important to note the great variability in the distribution of the number of cases in each procedure, ranging from 13 radical cystectomies to 159 transurethral removal of ureteral obstruction.

**Table 2:** Complications classified by unweighted severity grades

		**Severity grade**						**n**
**Procedure**	0*	**1**	**2**	**3**	**4**	**5**	**6**	
Inflatable penile prosthesis	13	2	1		2			18
Nephrourete rectomy	42	2	9	1	4	1		59
Partial nephrectomy	8		3		2			13
Percutaneous nephrostomy tube	31	1	1					33
Radical cystectomy	3	3	3	1	2	1		13
Radical prostatectomy	101	10	5	1	1			118
ESWL	33	2			1			36
Transurethral destruction of bladder lesion	32	2			4	1		39
Transurethral prostatectomy	56	15	5	1	10			87
Transurethral removal of ureteral obstruction	120	24	3		12			159
Ureteral catheterization	67	8	3	1				79
Subtotal by grade	506	69	33	5	38	3	0	654
Subtotal by grade (%)		47%	22%	3%	26%	2%	0%	

*“0” means no complications.

Table 3 (Additional file 2: Figure S1B) shows the complications classified by a weighted severity grade. By reporting these data, it was possible to understand the burden of complications in a given procedure and to compare the burden between two procedures that have similar PMIs. While Grade 1 complications made up 47% of the total complication (Table 2), it only accounted for 18% of the complication burden. The largest burden of complications was associated with Grade 4 complications, which comprised 26% of the total complications, but accounted for 53% of the complication burden. Grade 2 complications comprised 22% of the total complications and accounted for 20% of the complication burden. Grade 3, 5 and 6 complications were the least reported (total < 6%), accounting for a total complication burden of < 10%.

**Table 3:** Complications classified by weighted severity grades

	**Severity grade**						
**Procedure**	**1**	**2**	**3**	**4**	**5**	**6**	**Total**
Inflatable penile prosthesis	0.22	0.26		1.2			1.68
Nephroureterectomy	0.22	2.34	0.37	2.4	0.79		6.12
Partial nephrectomy		0.78		1.2			1.98
Percutaneous nephrostomy tube	0.11	0.26					0.37
Radical cystectomy	0.33	0.78	0.37	1.2	0.79		3.47
Radical prostatectomy	1.1	1.3	0.37	0.6			
ESWL	0.22			0.6			
Transurethral destruction of bladder lesion	0.22			2.4	0.79		3.41
Transurethral prostatetomy	1.65	1.3	0.37	6			9.32
Transurethral removal of ureteral obstruction	2.64	0.78		7.2			10.62
Ureteral catheterization	0.88	0.78	0.37				
Subtotal by grade	7.59	8.58	1.85	22.8	2.37	0	43.19
Subtotal by grade (%)	18%	20%	4%	53%	5%	0%	

Table 4 depicts the calculated PMI of the 11 reported procedures. As would be expected, a more complex procedure like radical cystectomy possessed a higher PMI (0.267), while a simpler procedure like percutaneous nephrostomy tube placement possessed a lower PMI (0.011). Thus the morbidity index associated with radical cystectomy was 24 times greater than the morbidity index associated with percutaneous nephrostomy tube placement. The PMI of the additional nine procedures fell within the range of the above PMIs. Table 4 also shows the complication rate and the severity of complication per case. For example, the complication rate of partial nephrectomy was about 38.46%, but if a complication occurs, its severity index was 0.396, which was about two times more severe than any complication that occurred during a ureteral catheterization.

**Table 4:** Postoperative morbidity index (PMI), complication rate and severity of complication by procedure

**Procedure**	**Complication rate (%)**	PMI*	Severity of complication**
Percutaneous nephrostomy tube	6.06	0.011	0.185
ESWL	8.33	0.023	0.273
Ureteral catheterization	15.19	0.026	0.169
Radical prostatectomy	14.41	0.029	0.198
Transurethral removal of ureteral obstruction	24.53	0.067	0.272
Transurethral destruction of bladder lesion	17.95	0.087	0.487
Inflatable penile prosthesis	27.78	0.093	0.336
Nephroureterectomy	28.81	0.104	0.360
Transurethral prostatectomy	35.63	0.107	0.301
Partial nephrectomy	38.46	0.152	0.396
Radical cystectomy	76.92	0.267	0.347

*Severity points per case where 0 = no complication and 1 death.

**Severity points per case with complication.

## Discussion

The volume of surgery over the past several decades has increased dramatically in all parts of the world, with an estimated 234 million operations performed annually, making safe delivery of surgical care a major public health concern [10]. From the early 1900’s, it has been a tenet of the surgical profession that the careful tracking and analysis of outcomes is essential to provide safe, high-quality care [1]. A simple, low-cost metric assessing post-operative complications, capable of providing rapid feedback to the surgical teams in any setting could therefore aid clinical care and quality improvement efforts.

The concept of severity weighting used to calculate the PMI is derived from utility weighting, which is the mathematical method of assigning value weights to multidimensional outcomes states to reflect overall impact [9]. The value of severity weights used in the current study comes from a well-validated study where 50 surgical experts were asked to evaluate and score 12 clinical vignettes [11]. Thus the PMI is an index, which might be most useful in detecting trends and serve as a point of reference in the surgical field. Considering this, the PMI numbers generated in the present study should be only taken as a starting point. For example, as shown in Additional file 1: Figure S1A, the PMI could be used to follow trends in complications for any particular urological procedure at the institutional level over months or years. As demonstrated in Table 3, when using the PMI, we are no longer simply analyzing the incidence rate of complications for a particular procedure, but we are also estimating the severity score of these respective complications. Furthermore, we can analyze the expected and actual severity grades of each complication that occurs. For instance, the post-operative complication rate of a transurethral prostatectomy was 35.63%, which could be further sub-classified into severity grades and compared to the complication grades with other procedures. This can be a valuable tool in standardizing practice or research, but also can be useful in properly counseling patients of their surgical risks. For example, one can advise that the severity of complications after a transurethral prostatectomy are approximately five times higher than the severity of a complication following a ureteral catheterization (PMI 0.10 *vs*. PMI 0.02, respectively).

It is evident that radical cystectomy at our institution had the most frequent complication rate and that most of the morbidity related to radical cystectomy was due to Grade 4 and Grade 5 complications, as shown in Additional file 1: Figure S1A. This high complication rate was noted by other investigators [12-14]. Specifically, DeNuzio and colleagues reported 415 complications in 302 patients undergoing cystectomy and classified these complications as Clavien type I (109 patients), II (220 patients), IIIa (45 patients), IIIb (22 patients), IV (11 patients) and V (8 patients) [14]. Furthermore, ureteral catheterization and ESWL had similar PMIs, but closer analysis shows that the burden of complications of ESWL came from Grade 4 complications, making it more severe than the burden from ureteral catheterization. Another important application of the PMI is to detect trends in deterioration or improvement in surgical outcomes, particularly after the institution of corrective measures or protocols. When analyzing this data and comparing with the curves in Additional file 1: Figure S1A, which displays the percentages of complications using the unweighted severity grades by procedure, similar PMI scores show completely different severity grade distributions. For example, when comparing nephroureterectomy and transurethral prostatectomy, both procedures have similar PMI scores (0.104 *vs.* 0.107), however transurethral prostatectomy has a greater number of grade 1 complications. In contrast, comparison of the curves in Additional file 2: Figure S1B, which shows the burden of weighted severity grades by procedure group, we can observe that most of the severity score for the transurethral prostatectomy is derived from grade 4 complications rather than from grade 1 as observed in the unweighted plot. In our series, we realized that almost all grade 4 complications in the transurethral prostatectomy were secondary to the need of a further resection of the prostate (data not shown). It would then be possible to further examine the indications for reoperation in our case series. Furthermore, this information can be used to compare surgeons, hospitals and procedures as well as initiate studies in order to determine the causes of such complications in that particular procedure. This technique of assessment then quality improvement could ultimately enhance the level of care provided to the urologic patient.

One limitation of this method is that it still lacks absolute objectivity in rating complications. For instance, there is no way to factor in whether the high reoperation rate after transurethral prostatectomy was due to the natural disease process of prostatic hypertrophy or to inadequate gland resection. Furthermore, we applied the severity weights of these ACS NSQIP 30-day morbidity derived from our general surgery colleagues. We believe these weights should be transferrable to the urologic patient, seeing that they are based on general medical/surgical tenets, but a follow-up study will validate these findings with urologic surgeons. Next, the patient population may have a higher comorbidity or some other factor that predisposes them to have a higher post-operative risk profile. These are important factors that must be factored into such a comprehensive system, however they are beyond the scope of this project. Thus, the technique utilized by our study of calculating PMI scores has limitations that must be considered before it can be applied in any institution or in any clinical situation. In concordance with Strasberg *et al.,* we believe that a) the simplicity of the PMI makes it an easy tool to implement but at the same time, a tool lacking the ability to perform individual risk adjustment, b) the fact that only the most serious complication in each patient is considered in order to calculate the PMI may tend to lose certain information when a patient presents with multiple complications, c) the PMI might be less useful for detecting differences across urological care providers at any point in time and d) the application of the PMI should be adequate for the majority of procedures, except for that ones with unusual complication, for example in the area of transplantation where the death of a living donor must receive a special severity weight.

## Conclusions

Based on the above results, quantitative severity weighing of post-operative complications of urologic procedures is feasible and may provide exceptionally informative data related to outcomes. As our national healthcare system continues to search for uniform yet applicable ways to measure and report quality care of the surgical patient, attention should be given to PMI.

## Abbreviations

PMI: Postoperative morbidity index; ACS: American college of surgeons; NSQIP: National surgical quality improvement program.

## Competing interests

The authors declare that they have no competing interests.

## Authors’ contributions

All authors have read and approved the final manuscript. JB, MD - Study concept and design, data collection, data analysis, drafting of manuscript. RS, MD – Study concept and design, data collection, data analysis, drafting of manuscript. DAP, MD, PhD – Statistical analysis and drafting of manuscript. CJR, MD, MBA - Study concept and design, drafting of manuscript. All authors read and approved the final manuscript.

## Pre-publication history

The pre-publication history for this paper can be accessed here:

http://www.biomedcentral.com/1471-2490/14/1/prepub

## Supplementary Material

Additional file 1: Figure S1A The burden of unweighted severity grades by procedure group (the y-axis is percentage of complication or the complication rate per 100 procedures). Click here for file

Additional file 2: Figure S1B The burden of weighted severity grades by procedure group (the y-axis is the complication rate per 100 procedures multiplied per the weighting factor). Click here for file
