# Supplementary figures and images for: The postoperative morbidity index: a quantitative weighing of postoperative complications applied to urological procedures
Source: BMC Urol. 2014 Jan 3;14:1. doi: 10.1186/1471-2490-14-1 (PMC3893398; doi:10.1186/1471-2490-14-1)

## Slide 1
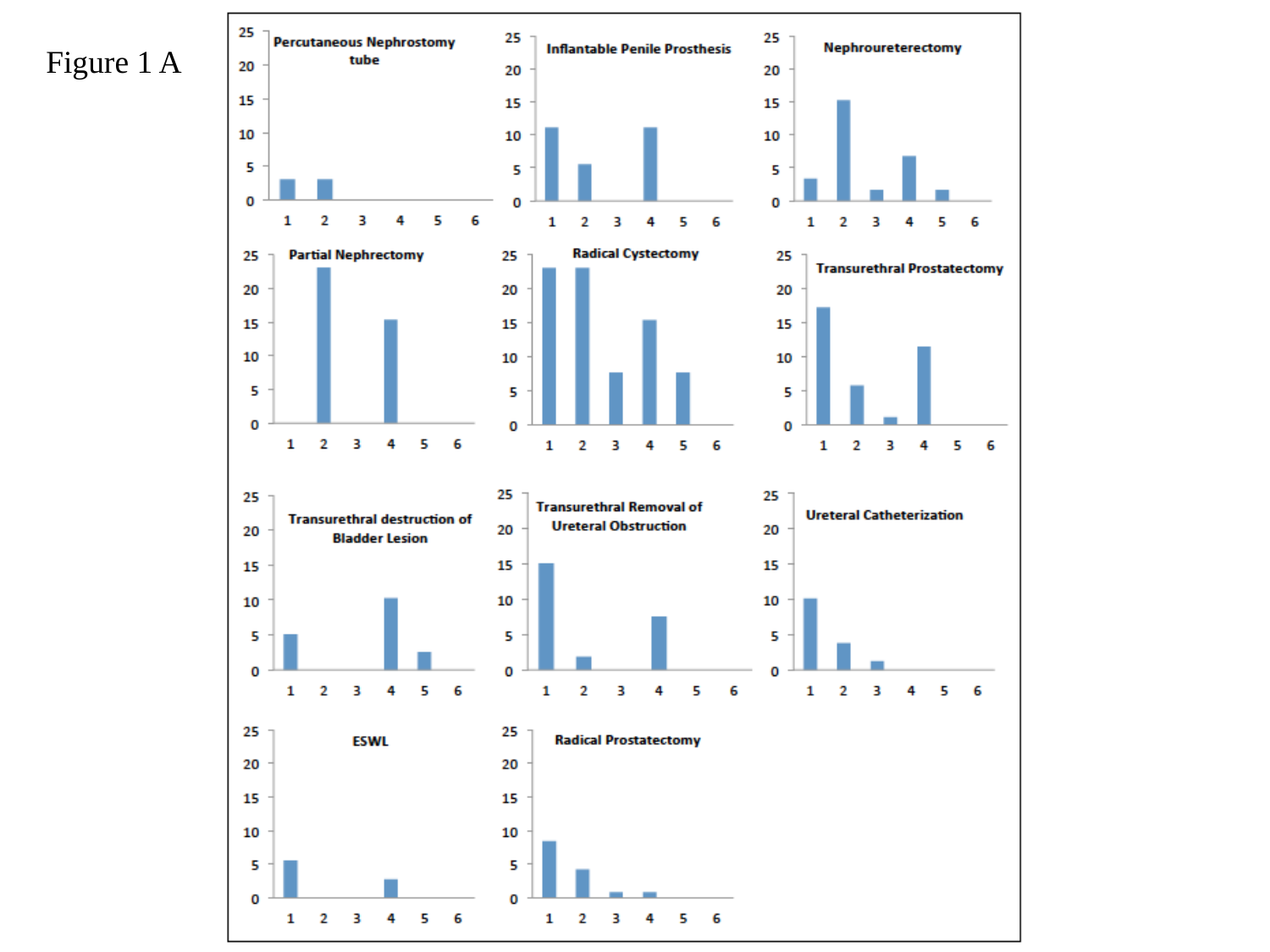

Figure 1 A

Supplement: Additional file 1: Figure S1A — The burden of unweighted severity grades by procedure group (the y-axis is percentage of complication or the complication rate per 100 procedures). [file 1471-2490-14-1-S1.pptx]

## Slide 1
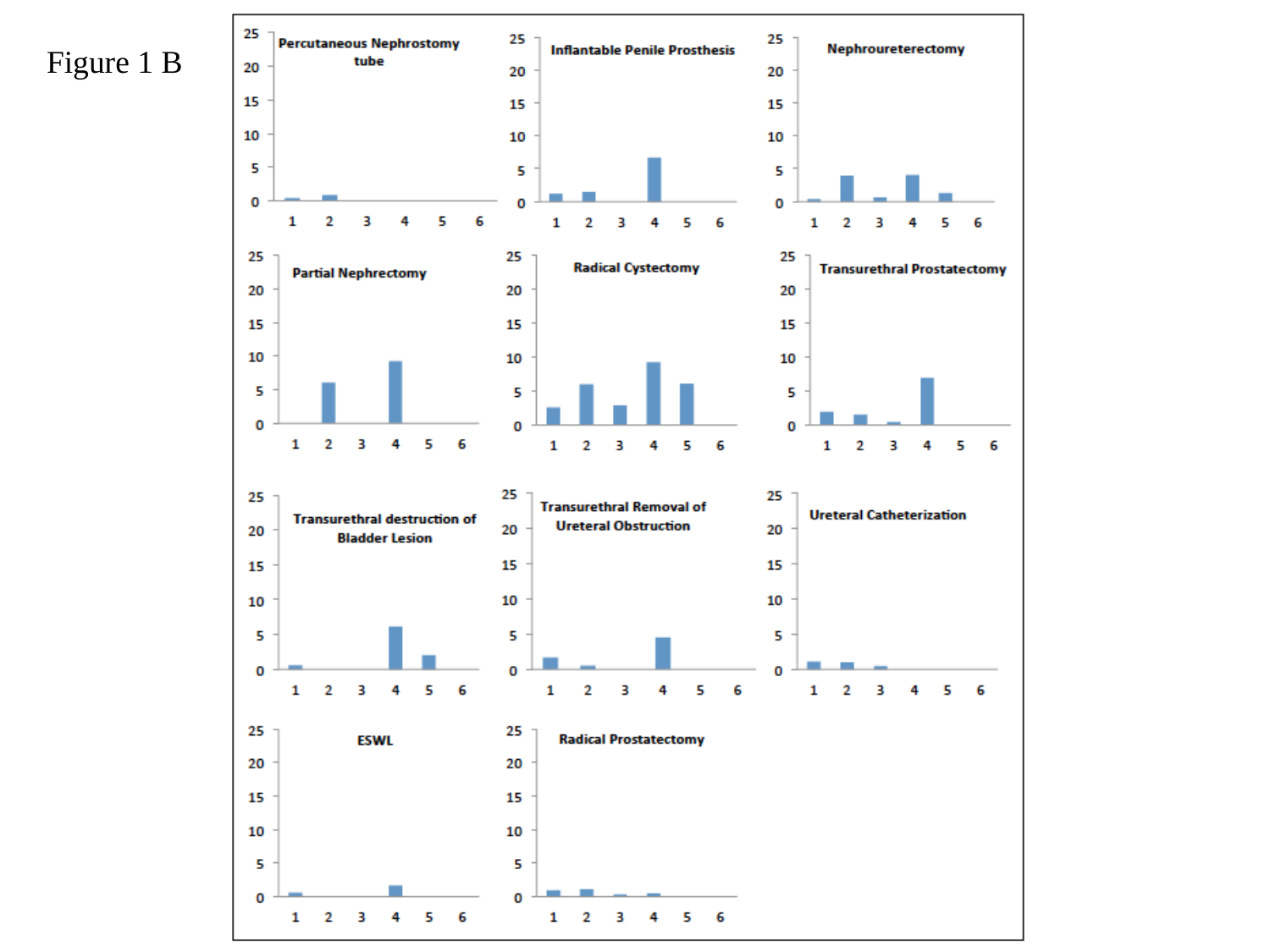

Figure 1 B

Supplement: Additional file 2: Figure S1B — The burden of weighted severity grades by procedure group (the y-axis is the complication rate per 100 procedures multiplied per the weighting factor). [file 1471-2490-14-1-S2.pptx]
